# Genetic Diversity and Population Structure of a *Camelina sativa* Spring Panel

**DOI:** 10.3389/fpls.2019.00184

**Published:** 2019-02-20

**Authors:** Zinan Luo, Jordan Brock, John M. Dyer, Toni Kutchan, Daniel Schachtman, Megan Augustin, Yufeng Ge, Noah Fahlgren, Hussein Abdel-Haleem

**Affiliations:** ^1^U.S. Arid Land Agricultural Research Center, Agricultural Research Service, United States Department of Agriculture, Maricopa, AZ, United States; ^2^Department of Biology, Washington University in St. Louis, St. Louis, MO, United States; ^3^Donald Danforth Plant Science Center, St. Louis, MO, United States; ^4^Department of Agronomy and Horticulture, University of Nebraska, Lincoln, NE, United States; ^5^Department of Biological and Agricultural Engineering, University of Nebraska, Lincoln, NE, United States

**Keywords:** *Camelina sativa*, population structure, genetic diversity, genotyping-by-sequencing (GBS), analysis of molecular variance (AMOVA)

## Abstract

There is a need to explore renewable alternatives (e.g., biofuels) that can produce energy sources to help reduce the reliance on fossil oils. In addition, the consumption of fossil oils adversely affects the environment and human health via the generation of waste water, greenhouse gases, and waste solids. *Camelina sativa*, originated from southeastern Europe and southwestern Asia, is being re-embraced as an industrial oilseed crop due to its high seed oil content (36–47%) and high unsaturated fatty acid composition (>90%), which are suitable for jet fuel, biodiesel, high-value lubricants and animal feed. *C. sativa*’s agronomic advantages include short time to maturation, low water and nutrient requirements, adaptability to adverse environmental conditions and resistance to common pests and pathogens. These characteristics make it an ideal crop for sustainable agricultural systems and regions of marginal land. However, the lack of genetic and genomic resources has slowed the enhancement of this emerging oilseed crop and exploration of its full agronomic and breeding potential. Here, a core of 213 spring *C. sativa* accessions was collected and genotyped. The genotypic data was used to characterize genetic diversity and population structure to infer how natural selection and plant breeding may have affected the formation and differentiation within the *C. sativa* natural populations, and how the genetic diversity of this species can be used in future breeding efforts. A total of 6,192 high-quality single nucleotide polymorphisms (SNPs) were identified using genotyping-by-sequencing (GBS) technology. The average polymorphism information content (PIC) value of 0.29 indicate moderate genetic diversity for the *C. sativa* spring panel evaluated in this report. Population structure and principal coordinates analyses (PCoA) based on SNPs revealed two distinct subpopulations. Sub-population 1 (POP1) contains accessions that mainly originated from Germany while the majority of POP2 accessions (>75%) were collected from Eastern Europe. Analysis of molecular variance (AMOVA) identified 4% variance among and 96% variance within subpopulations, indicating a high gene exchange (or low genetic differentiation) between the two subpopulations. These findings provide important information for future allele/gene identification using genome-wide association studies (GWAS) and marker-assisted selection (MAS) to enhance genetic gain in *C. sativa* breeding programs.

## Introduction

*Camelina sativa* (L. Crantz) originated from southeastern Europe and southwestern Asia and is a member of the Brassicaceae (Cruciferae) family, which contains a number of economically important crops such as *Brassica napus* (e.g., canola and rapeseed), *Brassica oleracea* (e.g., broccoli, cabbage, cauliflower) and *Brassica rapa* (e.g., turnip) (Singh et al., 2015). *C. sativa* was cultivated for food and oil since 4000 BCE in Scandinavia and Eastern Turkey (Berti et al., 2016), while genetic diversity studies have shown that Russia or the Ukraine are likely to be centers of origin (Sainger et al., 2017). *C. sativa* was displaced in the 1950s by canola, a higher-yielding oilseed crop, after being cultivated in Europe and North America for centuries. Public interest in *C. sativa* has been re-emerged recently due to its exceptional level of omega-3 essential fatty acids, favorable agronomic characteristics, and low-input potential as a biofuel crop (Ghamkhar et al., 2010). The oil content in *C. sativa* seeds (36–47%) can be up to twice that of soybean (18–22%) (Moser, 2012). The profile of *C. sativa* oil is low in saturated fatty acids (<10%) (Ghamkhar et al., 2010) and high in omega-3 α-linolenic essential fatty acids (up to 40% of total oil content) (Ghamkhar et al., 2010). These oil quality characteristics, combined with positive agronomic traits such as early maturity (Kagale et al., 2014), low-input requirements for water, nutrients, and pesticides (Manca et al., 2013; Kagale et al., 2014), broader adaptability to diverse environments (Singh et al., 2015), and resistance against insects and pathogens (Seguin-Swartz et al., 2009), make *C. sativa* an ideal alternative resource for biofuel and animal feedstock for the development of sustainable agriculture systems. However, since *C. sativa* fell out of favor until recently, few plant breeding and domestication efforts for the genetic improvement have been done. In addition, the availability of germplasm resources has also limited the breeding progresses. Currently, only scattered genetic resources were collected and stored at the European Catalogue of Plant Germplasm Collection^1^, the Plant Gene Resources of Canada database^2^, and the USDA-National Plant Germplasm System^3^.

Studies on genetic diversity and population structure are important for characterizing the natural selection history and genetic relationships among *C. sativa* accessions. The genome-wide assessments of the genetic landscape of *C. sativa* germplasm helps facilitate use of accelerated breeding approaches using marker-assisted selection (MAS). Previous works by other groups have yielded a reference genome resource for *C. sativa* and several relatively small-scale genetic studies. The reference genome (*n* = 20, genome size of ∼782 Mb) indicates an allohexaploid genome with three ancestral sub-genomes: two sub-genomes with seven chromosomes each derived from an older hybridization event that resulted in an allotetraploid ancestor, and a second hybridization between the tetraploid and a diploid ancestor that resulted in a sub-genome with six chromosomes (Kagale et al., 2014). The high degree of synteny and homologs found in *C. sativa* genome has high similarity and synteny with the *Arabidopsis thaliana*, which is a close relative in the *Camelineae* tribe of the *Brassicaceae* family (Berti et al., 2016). In addition to the reference genome, two genetic maps (Gehringer et al., 2006; Singh et al., 2015) were constructed and two small-scale genetic diversity studies (Vollmann et al., 2005; Ghamkhar et al., 2010) were conducted previously. These studies were based on relatively small populations (less than 100 accessions) from limited geographical regions (Ghamkhar et al., 2010), small numbers of molecular markers (Singh et al., 2015), or less advanced genotyping technology (e.g., AFLP and RAPD) (Vollmann et al., 2005; Gehringer et al., 2006; Ghamkhar et al., 2010). Therefore, to better characterize the current collection of the *C. sativa* diversity for future breeding efforts, a larger-scale population genetics analysis at the whole-genome level using advanced molecular genotyping strategies is needed.

The discovery and development of molecular markers has become progressively more rapid as next-generation sequencing (NGS) technologies become increasingly cost- and time-effective at the genome-wide level (Verma et al., 2015). Among all types of molecular markers, single nucleotide polymorphisms (SNPs) have been widely used due to their ubiquitous presence, uniform distribution, biallelic nature, and high heritability (Verma et al., 2015). Genotyping-by-sequencing (GBS) (Sonah et al., 2013) has proven to be an efficient high-throughput sequencing strategy for SNP discovery and genotyping in a single step (Davey et al., 2011) and has been widely applied to plant species such as *Brassica rapa* L. (Bird et al., 2017), *Ziziphus jujube* (jujube) (Chen et al., 2017), and *Triticum aestivum* L. (winter wheat) (Eltaher et al., 2018), and more. This strategy, when coupled with accurate and rapid phenotyping approaches, has the potential to considerably accelerate the genetic characterization of *C. sativa* germplasm, the estimation of phenotypic and genetic parameters, and the identification of marker-trait associations for the development of *C. sativa* as a domesticated crop.

In the present study, GBS technology was used to genotype a spring panel of 213 *C. sativa* accessions assembled from the Canadian germplasm collections in the USDA-ARS National Plant Germplasm System (NPGS) and the Leibniz Institute of Plant Genetics and Crop Plant Research (IPK). These accessions are originated from 19 different countries in Europe and Asia. The objectives were to (1) detect and genotype SNPs at a genome-wide scale; (2) characterize the genetic diversity and population structure; and (3) characterize genetic differentiation between and within the subpopulations. This study describes the genetic diversity and population structure in current *C. sativa* accessions and lays a foundation for future genome-wide association studies (GWAS) or genomic selection (GS) in *Camelina* breeding programs.

## Materials and Methods

### Plant Materials

**FIGURE 1:**
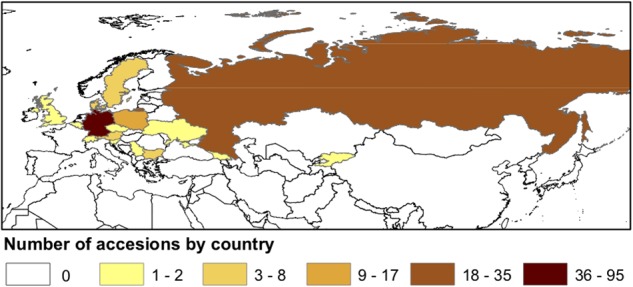
Geographical distribution of sampled *Camelina sativa* accessions.

A diversity panel of 213 *C. sativa* accessions, originally collected from different regions of Eurasia (Figure 1 and Supplementary Tables S1, S2), were assembled from the germplasm collections in the USDA-ARS NPGS and the Leibniz Institute of Plant Genetics and Crop Plant Research (IPK). ESRI ArcGIS v. 10.6 (Esri, 2011) was used to map accession density by country.

### DNA Extraction and Genotyping-by-Sequencing

*C. sativa* leaf tissue (∼0.13 g) was collected in Costar tubes on dry ice. The tissue tubes were stored in a rack and covered with breathable sealing tape and stored at −80°C until the tissue was lyophilized. *C. sativa* leaf tissue was ground in tubes with stainless steel beads using a plate shaker. DNA extraction on the lyophilized tissue was done using the Qiagen Plant DNeasy 96 kit following the manufacturer’s protocol. DNA concentration and quality was determined using Quantifluor (Promega, Inc.) and a Synergy H1 plate reader. The PstI restriction enzyme was used for GBS library construction (Elshire et al., 2011). Library construction and Illumina sequencing were done by the University of Cornell Genomic Diversity Facility.

### Sequencing Data Analysis

Raw sequence data was analyzed using the TASSEL v5.0 GBS v2 pipeline (Bradbury et al., 2007). An HTCondor Directed Acyclic Graph (DAG) workflow (Couvares et al., 2007) was used to run each step of the TASSEL pipeline. The HTCondor job files and DAG workflow are available at https://github.com/danforthcenter/camelina. Raw GBS sequencing data was converted to a unique tag database using the TASSEL GBSSeqToTagDBPlugin with a kmer size of 64 nucleotides and a minimum base quality score of 20 (kmerLength = 64, minKmerL = 20, mnQS = 20, mxKmerNum = 100000000). GBS tags were exported from the database in FASTQ format using the TASSEL TagExportToFastqPlugin and were aligned to the *C. sativa* genome using BWA MEM (Li and Durbin, 2009). Alignments in Sequence Alignment/Map (SAM) format were imported to the GBS database using the TASSEL SAMToGBSdbPlugin with settings such that all alignments get imported (aProp = 0.0, aLen = 0, minMAPQ = 0). SNPs were called from the imported alignments using the TASSEL DiscoverySNPCallerPluginV2 where sites had a minimum locus coverage across taxa of 0.1, a minimum minor allele frequency (MAF) of 0.01, and maximum of 64 tags allowed to align per cut site (maxTagsCutSite = 64, mnLCov = 0.1, mnMAF = 0.01). The TASSEL SNPQualityProfilerPlugin was used to calculate coverage, depth, and genotypic statistics for alignments in the database for all taxa. The TASSEL ProductionSNPCallerPluginV2 was used to export SNP data in Variant Call Format (VCF) (kmerLength = 64). *C. sativa* SNP were filtered to keep only biallelic sites with at most 20% missing data using vcftools (min-alleles = 2, max-alleles = 2, max-missing = 0.2) (Danecek et al., 2011). The VCF file was converted to HAPMAP format using the TASSEL export feature. The resulting SNPs were further filtered by disregarding the ones with MAF<0.05 for the following use.

### Population Genetic Analyses

#### Genetic Properties of Markers

The number of alleles and allele frequencies for the selected SNPs were calculated using vcftools (Danecek et al., 2011). The gene diversity (GD) of a locus, also known as its expected heterozygosity (*He*), is a fundamental measure of genetic diversity in a population, and describes the expected proportion of heterozygous genotypes under Hardy-Weinberg equilibrium (Nei, 1973). Formally, GD is the probability that a pair of randomly selected alleles from a population is different, and can be calculated as described by Harris and DeGiorgio (2017):

$$H=1-\sum_{i=1}^{I}P_{i}^{2}$$

where *I* is the number of distinct alleles at a locus and *P_i_* (*i* = 1,2, 3, …, I) is the frequency of allele *I* in the population. In addition to GD, MAF, and polymorphism information content (PIC) also indicate genetic properties of SNPs in a population from different aspects. MAF refers to the frequency at which the second most common allele occurs in a given population (Tabangin et al., 2009) and is computed as: MAF = the number of minor alleles in the population/total number of alleles in the population. Usually the SNPs with MAF smaller than 0.05 will be disregarded in most genetics studies. The PIC can be calculated using the following formula (Botstein et al., 1980):

$$\mathrm{PIC}=1-\sum_{i=1}^{n}P_{i}^{2}-\sum_{i=1}^{n-1}\sum_{j=i+1}^{n}2P_{i}^{2}P_{j}^{2}$$

where *P_i_* and *P_j_* are the frequencies of i^th^ and j^th^ alleles for the selected marker, respectively.

#### Analysis of Population Structure

Population structure was estimated using a Bayesian Markov Chain Monte Carlo model (MCMC) implemented in STRUCTURE v2.3.4 (Pritchard et al., 2000). Five runs were performed for each number of populations (k) set from 1 to 10. Burn-in time and MCMC replication number were both set to 100,000 for each run. The most probable *K*-value was determined by Structure Harvester (Earl and Vonholdt, 2012), using the log probability of the data [LnP(D)] and delta *K* (Δ*K*) based on the rate of change in [LnP(D)] between successive *K*-values. For the optimal *K*-value, membership coefficient matrices of five replicates from STRUCTURE were used in CLUMPP (Jakobsson and Rosenberg, 2007) to generate an individual Q matrix and a population Q matrix, which were then integrated with geographical location information (Supplementary Tables S1, S2) to create a bar plot using DISTRUCT software (Rosenberg, 2004). Accessions with membership probabilities greater than 0.5 were considered to belong to the same group. Genetic distances between pairs of accessions was calculated using GenAlEx v6.5 (Peakall and Smouse, 2012), from which a principal coordinate analysis (PCoA) was conducted. An unrooted neighbor-joining phylogenetic tree without the assumption of an evolutionary hierarchy was then constructed using the MEGA program (version 7.0) based on the obtained distance matrix, with 1,000 bootstrap replicates (Kumar et al., 2016). The principle behind this method is to construct a tree topology with pairs of neighbors that minimize the total branch length at each stage of neighbor clustering (Saitou and Nei, 1987).

#### Analysis of Molecular Variance (AMOVA) and Genetic Diversity Indices

The number of subpopulations determined with STRUCTURE were used for AMOVA and the calculation of Nei’s genetic distance in GenAlEx v6.503 (Peakall and Smouse, 2012). From AMOVA, the fixation index (Fst) and Nm (haploid number of migrants) within the population were obtained from GenAlEx v6.503 (Peakall and Smouse, 2012). Fst measures the amount of genetic variance that can be explained by population structure based on Wright’s F-statistics (Wright, 1965), while Nm = [(1/Fst) − 1]/4. An Fst value of 0 indicates no differentiation between the subpopulations while a value of 1 indicates complete differentiation (Bird et al., 2017). In addition, genetic indices such as number of loci with private allele, number of different alleles (*Na*), number of effective alleles (*Ne*), Shannon’s information index (*I*), observed heterozygosity (*Ho*) and expected heterozygosity (*He*) were also calculated using GenAlEx v6.503 (Peakall and Smouse, 2012).

## Results

### Characterization and Distribution of SNPs in the *Camelina sativa* Genome

**FIGURE 2:**
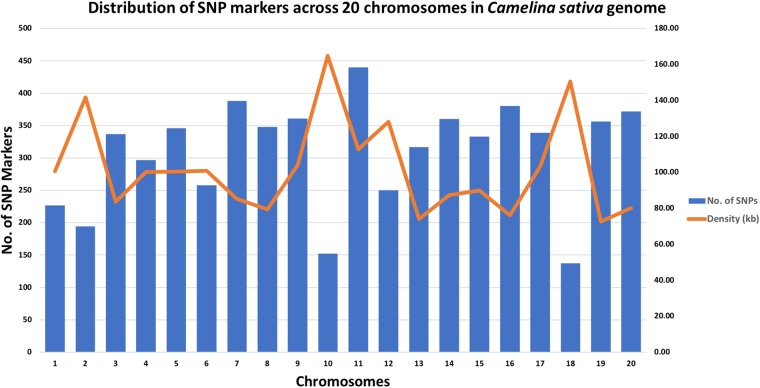
Genomic distributions of 6,192 SNPs across 20 *Camelina sativa* chromosomes and the corresponding SNP density.

A total of 213 *C. sativa accessions* were sequenced and genotyped using GBS. After sequencing data processing and SNP filtering, a total of 6,192 high-quality SNPs were physically mapped across 20 chromosomes with an average marker density of 101.77 kb per chromosome. A genome-wide SNP density plot (Figure 2) revealed that highest number of SNPs were physically mapped to chromosome 11 (7.1%, 440 SNPs). The highest and lowest marker densities were observed on chromosome 10 (164.73 kb) and chromosome 19 (72.59 kb), respectively (Figure 2 and Table 1). Transition SNPs (73.69%, 4,563 SNPs) were more frequent than transversions (26.31%, 1,629 SNPs), with a ratio of 2.80. The A/G transitions (37.24%) accounted for the highest frequency, while G/C transversions (4.47%) occurred at the lowest frequency among all the six SNP scenarios. The frequencies of two transition types were similar (i.e., A/G 37.24% and C/T 36.45%) while the frequencies of the four transversions types ranked as follows: A/T 8.04%, A/C 6.96%, G/T 6.83%, G/C 4.47% (Table 2).

**Table 1:** Genomic distribution of 6,192 SNPs mapped on 20 *Camelina sativa* chromosomes.

Chromosomes	No. of SNPs	% SNPs	Start position	End position	Length (Mb)	Density (Kb)
1	227	3.67	228704	23090767	22.86	100.71
2	194	3.13	69846	27568580	27.50	141.75
3	337	5.44	12546	28204286	28.19	83.66
4	297	4.80	155571	29874792	29.72	100.06
5	346	5.59	58645	34822707	34.76	100.47
6	258	4.17	363184	26361393	26.00	100.77
7	388	6.27	134626	33181162	33.05	85.17
8	348	5.62	47447	27676481	27.63	79.39
9	361	5.83	146807	37664901	37.52	103.93
10	152	2.45	89230	25128064	25.04	164.73
11	440	7.11	27486	49606425	49.58	112.68
12	250	4.04	268164	32316596	32.05	128.19
13	317	5.12	536996	24023072	23.49	74.09
14	360	5.81	127248	31599899	31.47	87.42
15	333	5.38	444993	30403961	29.96	89.97
16	380	6.14	92846	29000290	28.91	76.07
17	339	5.47	534721	35477318	34.94	103.08
18	137	2.21	199741	20820635	20.62	150.52
19	356	5.75	200572	26042767	25.84	72.59
20	372	6.01	36207	29870253	29.83	80.20

**Table 2:** Percentage of transition and transversion SNPs across the *Camelina sativa* genome.

SNP type	Transitions		Transversions			
	A/G	C/T	A/T	A/C	G/T	G/C
**Number of allelic sites**	2306	2257	498	431	423	277
**Frequencies**	37.24%	36.45%	8.04%	6.96%	6.83%	4.47%
**Total (percentage)**	4563 (73.69%)		1629 (26.31%)			

### Genetic Diversity (GD) and Polymorphism Information Content (PIC)

**FIGURE 3:**
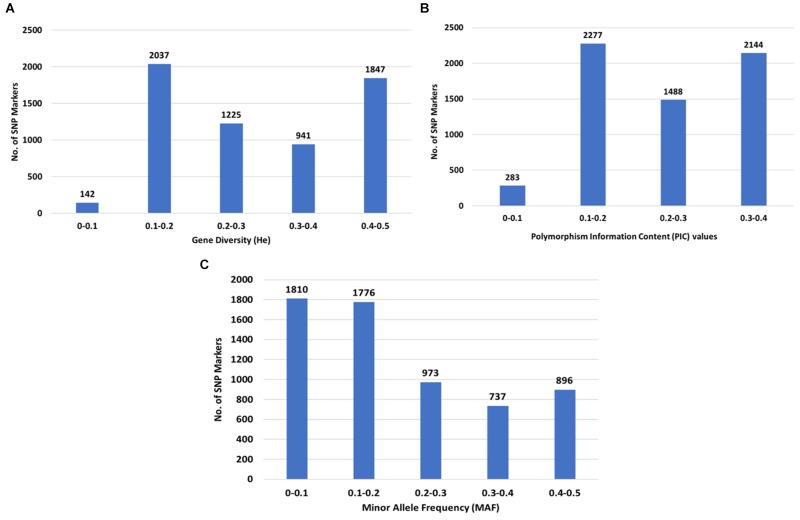
Distribution of genetic diversity for 6,192 SNP markers in the 213 *Camelina sativa* accessions. **(A)** Gene diversity (GD) or expected heterozygosity (*He*); **(B)** polymorphic information content (PIC); **(C)** minor allele frequency (MAF).

The GD values calculated as expected heterozygosity (*He*) in the population varied from 0.1 (142 SNPs) to 0.5 (1,847 SNPs) with an average of 0.29, while the PIC values varied from 0.1 (283 SNPs) to 0.4 (2,144 SNPs) with an average of 0.24 (Figure 3A,B). A total of 3,586 (57.9%) SNPs had a MAF less than 0.2 (Figure 3C).

### Population Structure and Genetic Relationships

**FIGURE 4:**
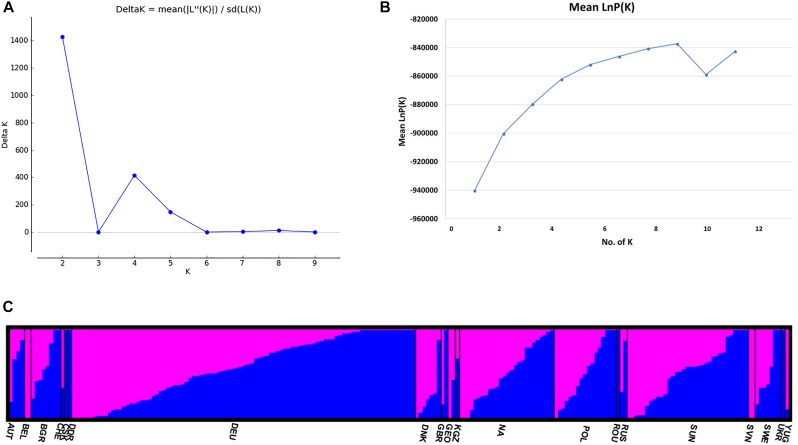
**(A)** Delta *K* (Δ*K*) for different numbers of subpopulations (*K*); **(B)** the average log-likelihood of *K*-value against the number of *K*; **(C)** estimated population structure of 213 *Camelina sativa* accessions on *K* = 2 according to geographical locations. *Accessions in blue were clustered into pop1 and the ones in pink were clustered into pop2*.

**FIGURE 5:**
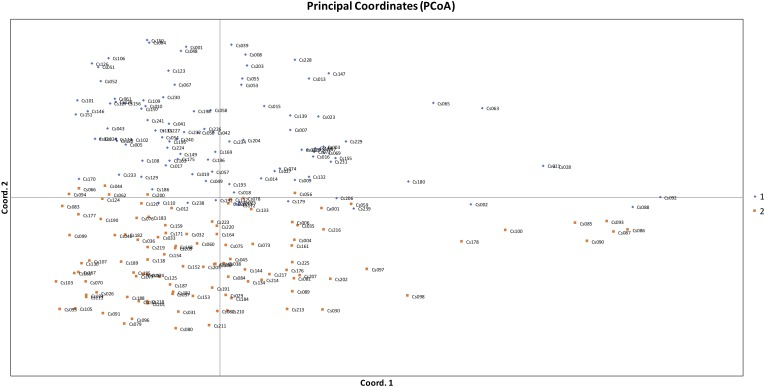
Principal coordinates analysis (PCoA) based on genetic distance showing two clustered subpopulations within studied *Camelina sativa* accessions.

The STRUCTURE v 2.3.4 (Pritchard et al., 2000) was used to study the population structure and genetic relations among the 213 *C. sativa* accessions that originating from 19 different countries in Europe and Asia (Supplementary Tables S1, S2). The *K*-value was used to estimate the number of clusters of the accessions based on the genotypic data throughout the whole genome. In order to find the optimal *K*-value, the number of clusters (*K*) was plotted against Δ*K*, which showed a sharp peak at *K* = 2 (Figure 4A). A continuous gradual increase was observed in the log likelihood [LnP(D)] with the increase of *K* except a slight decrease at *K* = 9 (Figure 4B). The optimal *K*-value indicates that two subpopulations (pop1 and pop2) showed the highest probability for population clustering and these two subpopulations consisted of 105 and 108 genotypes, respectively (Figure 4C, 5 and Supplementary Table S1). In addition, there was a small peak observed at *K* = 4 (Figure 4A), which might indicate another informative population structure. Therefore, the STRUCTURE results at both *K* = 2 and *K* = 4 were subject to the following population genetics analyses.

**FIGURE 6:**
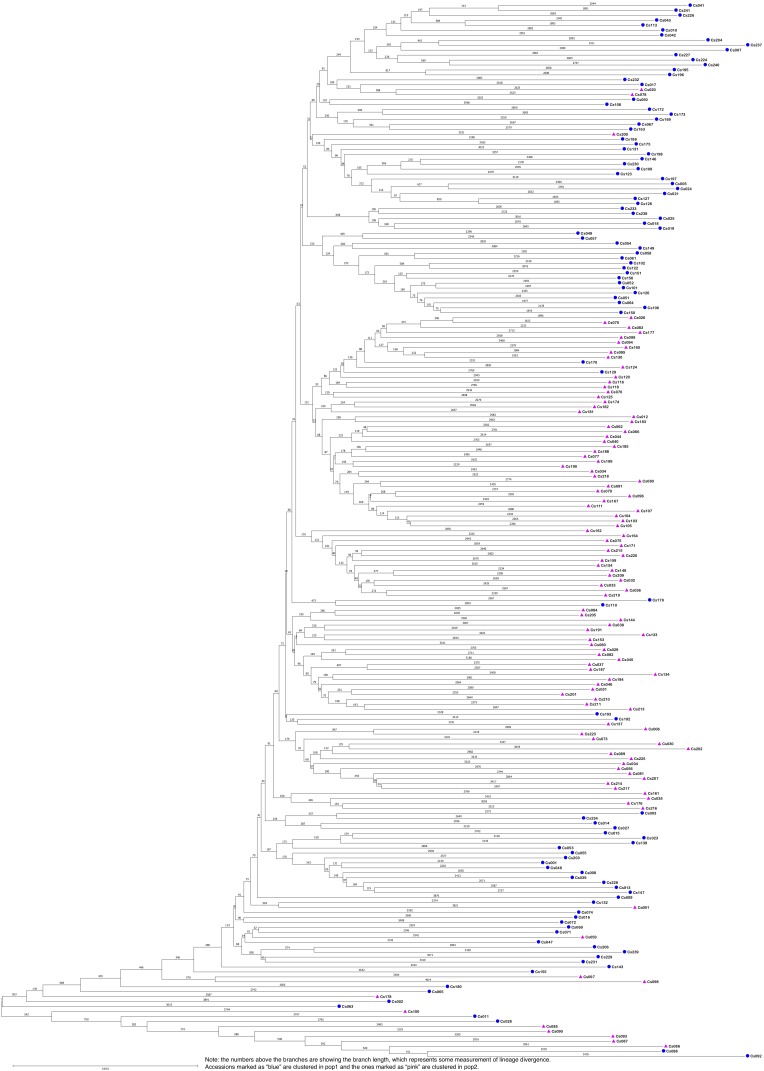
The neighbor-joining phylogenetic tree based on genetic distance matrix representing the grouping of 213 *Camelina sativa* accessions.

The STRUCTURE results estimated the fixation index (Fst) for each of the subpopulations and suggested that there was significant divergence within both of the two subpopulations (Table 3). An Fst value of 0.1612 and 0.2023 was obtained for pop1 and pop2, respectively (Table 3). In accordance with the STRUCTURE results, the principal coordinates analysis (PCoA) based on the pairwise genetic distance matrix among all the 213 C. sativa accessions also showed two clustered groups—one comprising 56.4% of accessions originating from Germany (DEU) and another consisting of 75.8% of accessions originating from Former Soviet Union (SUN) (Figure 4 and Supplementary Table S1). Additional STRUCTURE and PCoA results were provided for *K* = 4 (Supplementary Figures S1, S2 and Supplementary Table S3). A neighbor-joining phylogenetic tree (Figure 6) was constructed to represent the genetic distances among the population.

**Table 3:** The STRUCTURE results of 213 *Camelina sativa* accessions for the fixation index (Fst), average distances (expected heterozygosity/*He*) and number of genotypes assigned to each subpopulation.

Population	Inferred clusters	Mean Fst	Exp. Het	No. of genotypes
**Pop1**	0.468	0.1612	0.2749	105
**Pop2**	0.532	0.2023	0.2600	108

### Genetic Differentiation of Populations

The two subpopulations identified in STRUCTURE were then applied in GenAlEx 6.503 to calculate the Analysis of Molecular Variance (AMOVA), Nei’s genetic distance and the genetic diversity indices. The AMOVA, Fst and Nm are provided in Table 4. The AMOVA revealed that 4% of the total variation was found among subpopulations while the rest (96%) was within subpopulations. In addition, a high Nm (6.203) and a low Fst value (0.039) were obtained according to Nei’s genetic distance analysis. Further analyses were done on population structure at *K* = 4 and the results were shown in the Supplementary Tables S4, S5.

**Table 4:** Analysis of molecular variance (AMOVA) using 6,192 SNPs of the genetic variation among and within two subpopulations of 213 *Camelina sativa* accessions.

Source	df	SS	MS	Est. Var.	%
**Among pops**	1	12641.777	12641.777	50.300	4%
**Among individuals**	211	407210.434	1929.907	681.867	52%
**Within individuals**	213	120595.000	566.174	566.174	44%
**Total**	425	540447.211		1298.341	100%
**Fixation index (Fst)**	0.039				
**Nm (haploid)**	6.203				

### Allelic Pattern Across Populations

The grand mean value of different alleles (*Na*) and number of effective alleles (*Ne*) of the two subpopulations were 1.993 and 1.451, respectively (Table 5), and the mean value for the overall population in *I*, *He* and *uHe* were 0.438, 0.280 and 0.282, respectively. Pop1 (*I* = 0.449, *He* = 0.288, and *uHe* = 0.290) shows higher diversity than pop2 (*I* = 0.426, *He* = 0.272 and *uHe* = 0.274). The percentage of polymorphic loci per population (PPL) ranged from 98.74% (pop2) to 99.82% (pop1) with an average of 99.28%.

**Table 5:** Mean of different genetic parameters including number of samples (N), number of different alleles (Na), number of effective alleles (Ne), Shannon’s index (I), diversity index (h), unbiased diversity index (uh), and percentage of polymorphic loci (PPL) in each of the two subpopulations.

Pop	N	Na	Ne	*I*	*Ho*	*He*	*uHe*	F	PPL
**Pop1**	83.222	1.998	1.464	0.449	0.206	0.288	0.290	0.273	99.82%
**Pop2**	86.411	1.987	1.438	0.426	0.210	0.272	0.274	0.254	98.74%
**Mean**	84.817	1.993	1.451	0.438	0.208	0.280	0.282	0.263	99.28%

*N, the number of samples; Na, no. of different alleles; Ne, no. of effective alleles = 1/(Sum p_i_ˆ2); I, Shannon’s Information Index = −1^∗^ Sum [p_i_^∗^ Ln (p_i_)]; Ho, observed heterozygosity = no. of Hets/N; He, expected heterozygosity = 1 − Sum p_i_ˆ2; uHe, unbiased expected heterozygosity = [2N/(2N-1)] ^∗^ He; F, Fixation Index = (He − Ho)/He = 1 − (Ho/He), where p_i_ is the frequency of the i^th^ allele for the population and Sum p_i_ˆ2 is the sum of the squared population allele frequencies.*

## Discussion

To study the genetic diversity within *C. sativa*, a panel of 213 accessions was collected from IPK and USDA, which included 187 accessions originating from DEU (94), SUN (33), Poland (POL) (17), and 16 other countries in central Europe and Asia. The origins of 26 accessions were unknown (NA) (Supplementary Table S1). The genotypic data of the collected accessions was used for the investigation of genetic diversity and population genetics, which might underpin future breeding efforts (e.g., GWAS, etc.) in *C. sativa*.

### Genome-Wide SNP Discovery and Genotyping Using GBS

Consistent with previous studies involving *Camellia sinensis* (Yang et al., 2016), *Brassica napus* (Huang et al., 2013), and *Brassica rapa* (Park et al., 2010), transition SNPs were more frequent than transversions in *C. sativa*, indicating that transition mutations are better tolerated than transversion mutations during natural selection (Luo et al., 2017). This phenomenon is common on other plant species (Morton et al., 2006; Clarke et al., 2013; Mantello et al., 2014) and may be due to synonymous mutations in protein-coding sequences (Guo et al., 2017).

### Gene Diversity

Expected heterozygosity (*He*, also called gene diversity) and PIC values are both measures of genetic diversity among genotypes in breeding populations, which sheds the light on the evolutionary pressure on the alleles and the mutation rate a locus might have undergone over a time period (Botstein et al., 1980; Shete et al., 2000). The PIC values are a good indication of the usefulness of markers for linkage analysis when determining the inheritance between offspring and parental genotypes (Shete et al., 2000; Salem and Sallam, 2016), while GD (or *He*) indicates gene diversity for haploid markers and provides an estimate of the average heterozygosity and genetic distance among individuals in a population (Nei, 1990; Shete et al., 2000). In our study, the overall GD value was slightly greater than the PIC value (Figure 3), which was within our expectations since PIC values will always be smaller than GD (or *He*) and will become closer to GD with more alleles and with increasing evenness of allele frequencies (where it is less likely that individuals have identical heterozygote genotypes) (Shete et al., 2000). According to a previous study (Botstein et al., 1980), (1) markers with a PIC value ≧0.5 were considered to be highly informative; (2) markers with a PIC value from 0.25 to 0.5 were moderately informative; and (3) markers with a PIC value less than 0.25 were slightly informative. Our results showed that the PIC values for all the SNPs were less than 0.5, with an average PIC value of 0.24, suggesting that all the SNPs were considered moderately or low informative markers. Similar results were also found in winter wheat (Eltaher et al., 2018), *Lolium* spp. (ryegrass) (Roldan-Ruiz et al., 2000) and jujube (Chen et al., 2017). This may be due to the bi-allelic nature of the SNPs, which restricted PIC values to 0.5 (when the two alleles have identical frequencies) (Eltaher et al., 2018) and could also be due to low mutation rates in SNPs (Coates et al., 2009; Eltaher et al., 2018).

### Population Structure and Relationships

Population structure analysis is informative to understand genetic diversity and facilitates subsequent association mapping studies (Eltaher et al., 2018). The presence of population structure in the mapping population can lead to false positive associations between markers and traits (Eltaher et al., 2018). Therefore, testing the underlining population structure is the first step to conduct GWAS to identify a true association between markers and traits and the underlying genes controlling the traits. In our study, both the STRUCTURE results (optimal *K* = 2) (Figure 4A) and the PCoA results (Figure 5) indicated that the 213 *C. sativa* accessions could be clustered into two subgroups, and the PCoA results coincided with the STRUCTURE results. Moreover, the dendrogram analysis (neighbor-joining tree) gave similar results. The presence of structure in this population meets our expectation for the following reasons. First, according to the pedigree of genotypes (Supplementary Table S1), all the genotypes, although originally collected from 19 different locations in Europe, can be divided into two major geographical regions: one containing former SUN, Poland (POL), Russia (RUS), Slovenia (SVN), etc. and another one consisting of DEU, Denmark (DNK), Belgium (BEL), etc. Over 75% of accessions collected from SUN were clustered into the pop1 subgroup, as were all the accessions from RUS, SVN, and Sweden (SWE), and over 56.4% of accessions originated from DEU were clustered into the pop2, as were all the accessions originating from DNK, BEL, and United Kingdom (GBR). Secondly, certain specific traits intentionally selected by historic germplasm collectors or breeders might also lead to population structure. However, admixture of accessions between two subpopulations do exist, as was seen in Figure 4C and Supplementary Table S1. For example, 1 out of 8 Bulgaria (BGR) accessions and 41 out of 94 DEU accessions were clustered into pop1 while the majority were clustered into pop2. Likewise, 1 out of 4 Austria (AUT) accessions, 4 out of 17 Poland (POL) accessions and 8 out of 33 SUN accessions were clustered into pop2 while the majority were clustered into pop1. This might be due to genetic exchange among geographical regions, which were located close to or overlapping each other in eastern Europe and Asia. This admixture can also be expected from the similar threshold (pop1: 0.468, pop2: 0.532) when accessions were grouped into inferred clusters from STRUCTURE software, resulting in a small number of accessions clustered completely into a certain group while the majority of them can be somewhat clustered into both groups (Figure 4C). Nevertheless, due to the limitations of the amount of collected accessions and the extensity of the geographical origins, for most of origins, there are only a few accessions assigned (Supplementary Table S1), resulting in possible uncomprehensive and unassured speculation for genetic exchange. Moreover, as for a relatively recent domesticated plant species like *C. sativa*, much of its varietal diversity was lost in the 20th century when European farmers shifted their interest from the cultivation of *C. sativa* to rapeseed, sunflower and other species for oilseed production (European Commission, 2017), and the current publicly available germplasm collections are almost entirely composed of previous cultivated varieties (Brock et al., 2018), therefore, it’s not surprising that a low genetic diversity and a high proportion of admixture are exist. Similar results have also been found in previous *Camelina* breeding lines and cultivars (Vollmann et al., 2005), which were mainly collected from the Russia-Ukraine region that is a common origin area of *C. sativa*. Brock et al. (2018) found a low genetic diversity among *C. sativa* accessions. However, our result contradicted Ghamkhar et al. (2010) study that indicated a high genetic diversity using AFLP fingerprinting of 53 accessions collected from Russian-Ukrainian region. Maybe the low sample size in their study resulted in a relatively biased conclusion.

### Genetic Differentiation of Populations

Fst is a measure of population differentiation due to genetic structure. An Fst value greater than 0.15 can be considered as significant in differentiating populations (Frankham et al., 2002). Thus, a significant divergence was found within each of the *C. sativa* two subpopulations according to the Fst values obtained from the STRUCTURE (Table 3). However, a low Fst value (0.039) was found between the two subpopulations (Table 4), indicating a low genetic differentiation between these two subpopulations. This coincided with the AMOVA results (Table 4), where the vast majority of total variation (96%) was accounted for by within-subpopulation variations while only 4% of total variation was accounted for by among-subpopulation variations. Wright (1965) reported that a Nm value less than one indicate limited gene exchange among subpopulations while in our study, the Nm value of 6.203 was high, suggesting that a high genetic exchange or high gene flow (Eltaher et al., 2018) may occur and led to a low genetic differentiation between subpopulations.

Undeniably, the STRUCTURE results showing another peak at *K* = 4 (Figure 4A and Supplementary Table S1) may suggest another informative population structure. However, the low Fst values among subpopulations (Supplementary Table S3), the low Nei’s genetic distance (Supplementary Table S4), AMOVA results (Supplementary Table S5) as well as the confounding PCoA results (Supplementary Figure S2) didn’t show a better separation of the subpopulations than *K* = 2. It is not surprising that several clustered populations could appear to be informative to represent the actual population structure after the STRUCTURE analysis, since sometimes the population within certain geographical regions may be variable and a genetic structure may already exist or the species may be structured into ecotypes or host races due to gene flow or common ancestry even if it spread across different geographical regions (Meirmans, 2015). It is difficult and not necessary to hierarchically structure the populations in different levels. For example, a previous finding showed that *C. sativa* was descended from its pre-domesticate species *C. microcarpa* due to their similar genome size and low genetic differentiation between the two species (Brock et al., 2018). This could be one of the explanations for the peak at *K* = 4 (Figure 4A) and admixture proportions between the subpopulations as shown in the PCoA results (Supplementary Figure S2). Similar phenomenon has also been seen in other researches (Giri et al., 2017; Zhao et al., 2018; Zhou et al., 2018).

### Allelic Pattern and Genetic Diversity Indices

The allelic pattern and genetic diversity indices provided insight to genetic diversity within each of the two subpopulations. Although both subpopulations had similar expected heterozygosity (*He*), pop1 was slightly higher than pop2, meaning that pop1 was more diverse than pop2 since *He* depends on both the number of alleles (richness) and the abundance (or evenness) of the alleles in a population. The low genetic diversity and the clusters of two subpopulations were in agreement with a previous population genetics study among a collection of 175 accessions of *C. sativa* (Singh et al., 2015) using 493 SNPs. The understanding of genetic diversity within *C. sativa* populations will enhance future planning in breeding programs and provide helpful information in maintaining and monitoring genetic diversity required for a robust breeding program (Eltaher et al., 2018).

## Conclusion

In this study, high-throughput GBS technology was used to explore genetic diversity and population structure among the current *C. sativa* accessions and the possibility of using SNP markers for genomic analyses in genetic enhancement. Based on our data, the panel was genetically diverse. This level of genetic diversity could be the basis for developing new *Camelina* cultivars with desirable characteristics such as high yield potential, high oil production and tolerance to abiotic stress while being adapted to diverse environments. Moreover, our study identified two subpopulations which could be explained by their geographical differentiation, natural selection and regional adaptation history. The pop1 is more diverse than pop2 based on Shannon’s information index (*I)*, expected heterozygosity (*he)*, unbiased expected heterozygosity (*uhe)*, and percentage of polymorphism loci (PPL). This knowledge of population structure and genetic diversity of *C. sativa* accessions will be important for future studies using genomic selection, MAS and GWAS.

## Author Contributions

HA-H and NF conceived and designed the study and provided suggestions and comments for the manuscript. NF performed GBS analysis. ZL collected and analyzed the data and wrote the manuscript. ZL, JB, JD, TK, MA, DS, YG, NF, and HA-H revised the manuscript. All authors read and approved the manuscript.

## Conflict of Interest Statement

The authors declare that the research was conducted in the absence of any commercial or financial relationships that could be construed as a potential conflict of interest.

## Footnotes

1 http://eurisco.ecpgr.org

2 http://pgrc3.agr.gc.ca/search_grinca-recherche_rirgc_e.html

3 https://www.ars-grin.gov

## Supplementary Material

FIGURE S1 Estimated population structure of 213 Camelina sativa accessions on *K* = 4.

FIGURE S2 Principal coordinates analysis (PCoA) based on genetic distance showing four clustered subpopulations within studied Camelina sativa accessions.

TABLE S1 Geographical distribution of 213 *Camelina sativa* accessions.

TABLE S2 The number of *Camelina sativa* accessions originated from 19 different countries in Europe and Asia.

TABLE S3 Pairwise Fst values among four subpopulations.

TABLE S4 Nei’s pairwise genetic distance among four subpopulations.

TABLE S5 Analysis of Molecular Variance (AMOVA) using 6,192 SNPs of the genetic variation among and within four subpopulations of 213 *Camelina sativa* accessions.
