# Supplementary figures and images for: Genetic Diversity and Population Structure of a Camelina sativa Spring Panel
Source: Front Plant Sci. 2019 Feb 20;10:184. doi: 10.3389/fpls.2019.00184 (PMC6391347; doi:10.3389/fpls.2019.00184)

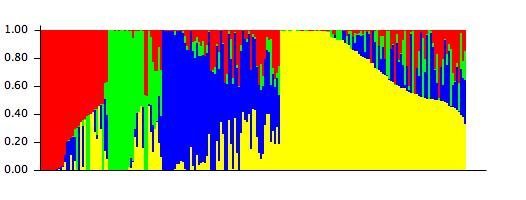

Supplement: FIGURE S1 — Estimated population structure of 213 Camelina sativa accessions on K = 4. [file Image_1.tif]

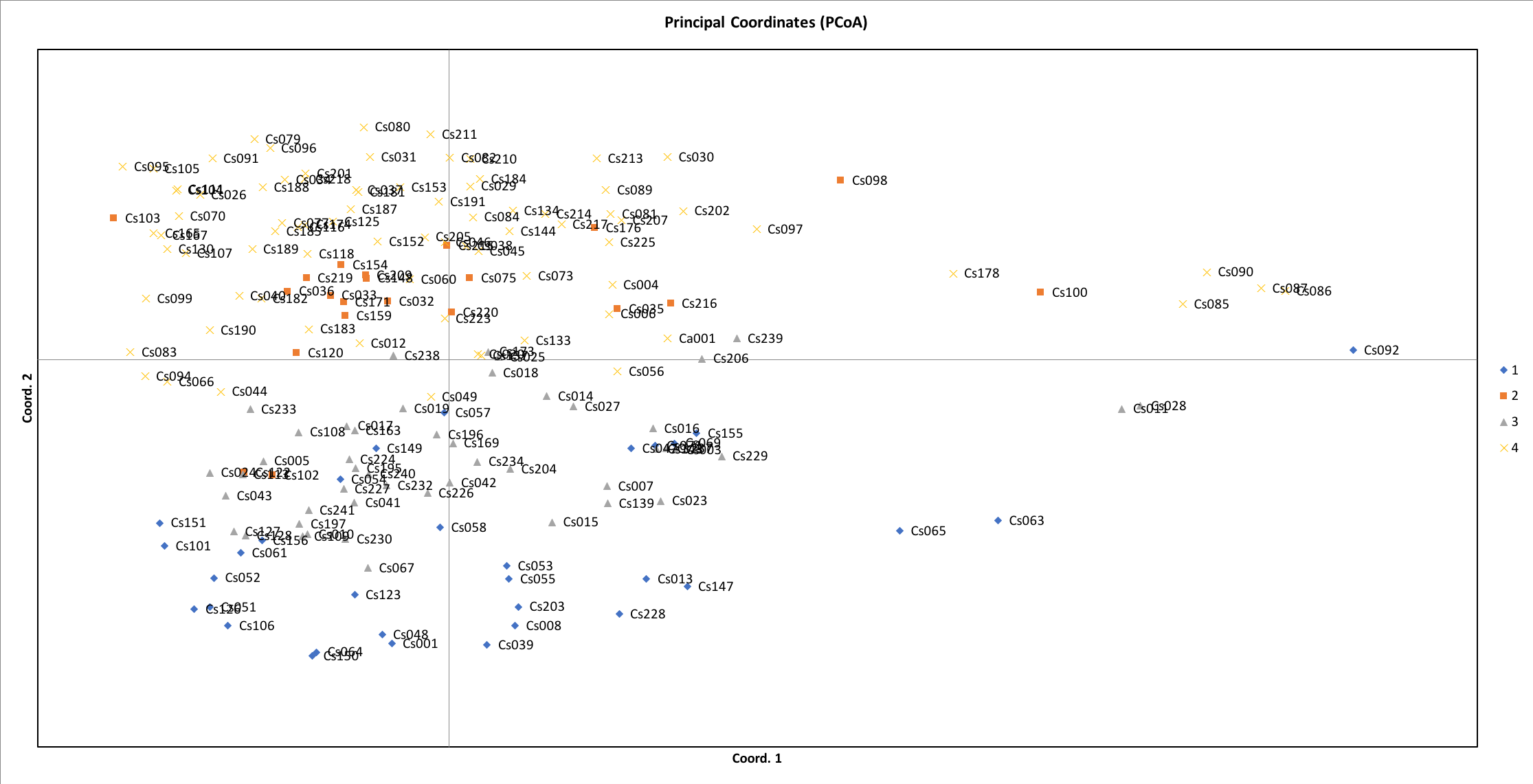

Supplement: FIGURE S2 — Principal coordinates analysis (PCoA) based on genetic distance showing four clustered subpopulations within studied Camelina sativa accessions. [file Image_2.tif]
